# Human Milk Practices in Swedish Neonatal Units: Results From a Nationwide Survey

**DOI:** 10.1111/apa.70448

**Published:** 2026-01-20

**Authors:** Ylva Thernström Blomqvist, Anna‐My Lund, Fredrik Ahlsson, Jenny Ericson, Charlotta Karlsson, Josefin Lundström, Ingela Sandström, Anna Gustafsson

**Affiliations:** ^1^ Neonatal Intensive Care Unit University Children's Hospital Uppsala Sweden; ^2^ Department of Women's and Children's Health Uppsala University Uppsala Sweden; ^3^ Department of Clinical Sciences, Paediatrics Skåne University Hospital, Lund University Lund Sweden; ^4^ Department of Health and Welfare Dalarna University Falun Sweden; ^5^ Department of Clinical Microbiology and Infection Prevention and Control Kronoberg Region, Central Hospital Växjö Sweden; ^6^ Department of Neonatology Sachsska Children and Youth Hospital Stockholm Sweden; ^7^ Department of Women's and Children's Health Karolinska Institutet Stockholm Sweden

**Keywords:** donor human milk, human milk, infant, mother's own milk, neonatal nutrition practice, neonatal unit, preterm

## Abstract

**Aim:**

The aim of this study was to describe the routines for handling mother's own milk and donor human milk in Swedish neonatal care units related to national guidelines.

**Methods:**

A web‐based survey of 24 of 38 neonatal units and 19 of 28 human milk banks in Sweden.

**Results:**

Despite national guidelines, breast milk handling varied widely. All units offered lactation support and free breast pumps during hospital stays; nine continued post‐discharge. Use of mother's milk differed; some prioritised fresh, others mixed or used frozen milk by age. Donor milk was mainly given to infants < 34–35 weeks or post‐surgery, though criteria varied. Some also provided it for hypoglycaemia, growth restriction or hypothermia treatment. Fortification routines varied in both target groups and preparation, ranging from bedside at each feed to once daily. Most milk banks tested donor milk before pasteurisation; one tested both before and after. Five units never declined donations, while others did due to storage limits, surplus supply or time constraints.

**Conclusion:**

Substantial variation in practices indicates a need to clarify and update national guidelines and strengthen milk banking, with relevance both nationally and internationally.

## Summary

- This study highlights variations in practices at neonatal units and Swedish HMBs regarding routines for mother's own milk and donor human milk handling and administration.
- There is a need to facilitate milk donation and increase the capacity of human milk banking in Sweden.
- There is a need for improved standardisation, clarification and implementation of guidelines at the national level.

**Abbreviations**
DHM: donor human milk
ESBL: extended spectrum beta lactamase
HIV: human immunodeficiency virus
HMB: human milk bank
HTLV: human T‐lymphotropic virus
MOM: mother's own milk
MRSA: meticillin‐resistant *Staphylococcus aureus*

NEC: necrotizing enterocolitis
NSAID: non‐steroidal anti‐inflammatory drugs
VLBW: very low birth weight

## 1 Background

Human milk is recognised as the ideal nutrition for all newborns, including those needing specialised care in neonatal units. Mother's own milk (MOM) is the primary option [1]; however, when breastfeeding or MOM is unavailable, donor human milk (DHM) is recommended as the second‐best choice for infants born preterm and infants needing neonatal care [2, 3, 4]. Even though both MOM and DHM are defined as human milk, there are some differences due to donor lactation state and the processing of donor milk [5, 6]. In most countries, DHM is pasteurised, reducing or eliminating many of the bioactive factors in human milk [7].

Breast milk provides all the energy and essential nutrients that a healthy newborn requires during the first six months of life [8]. Antibodies transferred through the breast milk also play an important role in the early protection of newborn infants [9]. For preterm infants, human milk provides protection against diseases such as necrotizing enterocolitis (NEC) [10]. Human milk enhances cognitive development in preterm infants [11] and reduces the risk of various illnesses, including sepsis, respiratory infections and urinary tract infections [12]. Additionally, infants in neonatal units are more likely to achieve full enteral feeding volumes when human milk, instead of formula, is provided, which can help reduce the duration of parenteral nutrition [13]. Moreover, when human milk is provided to preterm infants, the risk of developing high blood pressure later in life is decreased, reducing the risk of cardiovascular diseases [14, 15, 16]. In neonatal units, MOM is during the initial period mainly provided through maternal breast milk expression, often by using a breast milk pump. Research shows that initiating breast milk expression within six hours of an extremely preterm birth is associated with higher overall milk production and greater yield per expressing session [17]. To promote timely initiation of breast milk expression, evidence based information is ideally provided to both parents during a prenatal consultation with the neonatology team. The importance for the infant's health of providing MOM as soon as possible after birth should be emphasised. Many Swedish neonatal units provide a so‐called colostrum kit, which includes detailed instructions and practical guidance for the early initiation of breast milk expression to support the mother in this process [18, 19]. A colostrum kit includes written information about factors associated with milk production, how to perform milk expression, as well as devices to collect the expressed milk. A national network, Milknet, was created in 2001, providing guidelines [20] and support to the HMBs and neonatal units. Ahead of an upcoming update of guidelines, a survey of existing routines was carried out.

### 1.1 Aim

The aim of this study was to describe the current routines regarding MOM and DHM in Swedish neonatal units and HMBs related to national guidelines.

## 2 Methods

### 2.1 Setting

There are currently 38 neonatal units in Sweden (Appendix S1). All neonatal units have access to a so‐called ‘milk‐kitchen’, a facility in which MOM, DHM and formula, including fortification of these when required, are prepared and stored. The definition of a HMB in this study is a facility that handles all parts of the DHM processes, including pasteurisation. The HMBs in Sweden are responsible for the processing of DHM and are hospital‐based and managed by staff from the neonatal unit or staff from other departments. Neonatal units without a HMB in their hospital collaborate regionally to access DHM.

### 2.2 Survey Design and Data Collection

**FIGURE 1:**
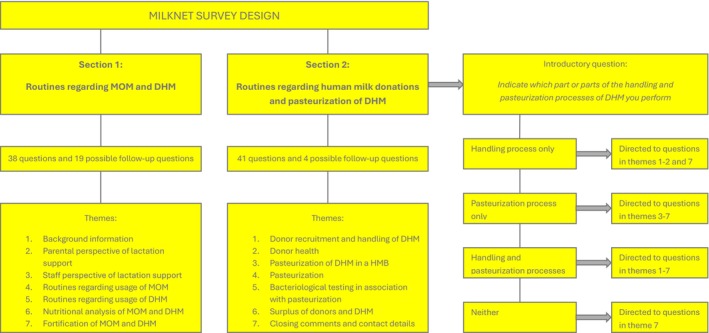
Survey design.

The Milknet board developed a web‐based survey with a total of 102 questions designed as either single‐choice questions, multiple‐choice questions or open‐ended questions (Appendix S2). In total, the survey included 23 possible follow‐up questions and 79 primary questions. The survey was based on the current Milknet guidelines [20], the scientific literature and clinical experience. The survey design, including the investigated themes, is illustrated in Figure 1. An invitation email with a web‐link to the survey was sent in November 2022 to all neonatal units in Sweden.

### 2.3 Response Rate

Not all questions were answered in each survey. In most cases, the survey was completed by several professions, for example, physicians, registered nurses, assistant nurses, midwives, nutrition assistants, biomedical analysts and department heads. Only two surveys were completed by a single professional. For organisational reasons, three neonatal units gave joint responses; hence, the maximum number of responding neonatal units was 36 rather than 38. The survey was completed by 24 out of 36 units, resulting in an overall response rate of 67%. The included neonatal units represented all six healthcare regions in Sweden (Table 1).

**TABLE 1:** Characteristics of participating units.

	*n*/*N*
Level of care provided^a^	24/24
Neonatal intensive care	17
Neonatal care	23
Couplet care	14
Neonatal home care	19
Neonatal follow‐up clinic	18
Provision of neonatal care from birth: gestational age criteria (weeks + days)	24/24
22 + 0	4
28 + 0	12
29 + 0	3
30 + 0	3
32 + 0	1
35 + 0	1
National health care region	24/24
South (*N* = 8)	5
South‐east (*N* = 5)	4
West (*N* = 4)	4
East (*N* = 6^b^)	2
Middle (*N* = 8)	6
North (*N* = 7)	3
Donor milk processes	24/24
Handling process only	5
Pasteurisation process only	0
Handling and pasteurisation processes	19
Location of human milk bank	19/19
Neonatal ward	15
Own premises outside ward	4

^a^ Multiple answer possible.

^b^ Three hospitals have the same organization and gave a joint answer.

### 2.4 Data Analysis

Data were analysed using descriptive statistics; due to the small sample size, only numbers are presented. A simplified form of qualitative content analysis [21] was used for the follow‐up answers in free text, and its results are presented descriptively.

### 2.5 Ethical Considerations

The study was conducted in accordance with the Declaration of Helsinki and was approved by the Swedish Ethical Review Authority (Dnr 2022‐01841‐01).

## 3 Results

Background information regarding the responding neonatal units, including which parts of the donor milk processes the units are involved in, is presented in Table 1. In all neonatal units, parents could be together with their infant 24/7.

### 3.1 Staff Training Regarding Lactation and Breastfeeding

Regular staff training regarding lactation and breastfeeding was out of the 24 responding units reported as follows: 13 units provided training to all newly hired staff, 10 units provided training to all staff once a year, one unit provided training to all staff every second year, seven units provided training to all staff more seldom, and two units did not offer any training at all. Nine of the 23 units offered the training to all professions working in the neonatal unit. Twenty‐one units reported that the training was provided by internal staff, seven units collaborated with the associated university for these training sessions and one unit sought assistance from industry.

### 3.2 Lactation Support and Parental Education

All units offered lactation support (Table 2), the staff providing this support was primarily from the neonatal unit but also from the maternity ward. Information was provided regarding differences between MOM and DHM: however, two units reported that they did not educate the parents regarding differences between MOM and DHM and four units stated that they only did so occasionally. All units except one followed up on maternal milk expression during the hospital stay.

**TABLE 2:** Lactation support and parental education.

	*N* = 24
	*n* (missing^a^)
Staff providing lactation support and education for parents	24
Assistant nurses (college‐level education in nursing)	24
Registered nurses	22
Midwives	9
Dietitian	0
Medical doctors	8
Other	5^a^
Lactation consultant available	1
Units with written guidelines for lactation support	
Yes	11 (1)
No	12 (1)
Content of education and support provided to parents	
Importance of breast milk	24
Yes	21
Sometimes	3
Difference between MOM and DHM	24
Yes	18
No	2
Sometimes	4
Using Colostrum‐kit as a tool	24
Yes	16
Sometimes	3
No	5
When is the Colostrum‐kit provided to mothers?	19
Distributed before birth	5
Distributed after birth	8
Could be distributed either before and/or after birth	8
Follow‐up provided on mother's milk expression	24
Yes	23
No	1
Follow‐up on mother's milk expression provided by	23
Assistant nurses (college‐level education in nursing)	22
Registered nurses	23
Midwives	5
Dietitian	0
Medical doctors	5
Other	5^b^
Follow‐up on mother's milk expression includes	23
Maternal emotions on milk expressions	18
Expressed milk volume	17
Other	11^c^
How often is follow‐up on mother's milk expression provided	23
Daily	17
< 3 times a week	3
Once a week	3
Facilitating lactation in the neonatal unit and after discharge	
Provision of meals to lactating mothers of infants cared for in the neonatal ward	24
Yes	7
No	17
Which meals are provided to lactating mothers of infants cared for in the neonatal ward	7
Breakfast	7
Lunch	5
Evening meal	6
Milk expression privacy	24
Mothers could express privately at the hospital	19
Mothers could occasionally express privately	5
Provision of breast pumps for free during hospital stay	24
Electric breast pump	24
Manual breast pump	7
Provide electric breast pumps after discharge	24
Free of charge	9
For a fee	15^d^

^a^ Free text answers were midwives' responsibility and experienced (not new) nurses, assistants with a high‐school degree in Children and Leisure studies (*n* = 3), specific staff in a breastfeeding group in the neonatal ward, and a lactation consultant.

^b^ Free text answers were assistants with a high‐school degree in Children and Leisure studies, physicians, nutrition assistants and lactation consultants.

^c^ Free text answers were pumping frequency, support to pump often, mothers' feelings of time for pumping, right size of shield, practical information, used pumping programme, according to individual needs, support for maternal rest and food intake.

^d^ Free text answers, 11 units reported free of charge while in home care but for a fee after discharge from home care, three units reported free of charge if the mother was a milk donor and three units reported free of charge if certain diagnoses in the mother or infant.

Other factors that may facilitate lactation in the neonatal unit and after discharge may be provision of meals for mothers and breast pumps. Seven of 24 units provided meals for mothers during neonatal care.

### 3.3 Routines Regarding Mother's Own Milk

#### 3.3.1 Prioritisation of Fresh or Frozen MOM to the Infant

Nineteen of the 24 responding units reported that when MOM was frozen it was provided to the infant in chronological order, that is, frozen MOM with older expression dates were used before frozen MOM with more recent expression dates. In cases where both freshly expressed MOM and previously expressed frozen MOM were available, four of the 24 responding neonatal units prioritised providing the infant with frozen MOM in chronological order, 10 units prioritised giving fresh MOM and the remaining 10 units prioritised providing the infant with a combination of fresh and frozen MOM. In the written responses one unit clarified prioritising fresh MOM over frozen; however, they opted for the frozen MOM if it contained more energy/fat. Two of the units that prioritised providing frozen MOM made an exception during the first postnatal week to provide the infant with fresh colostrum.

#### 3.3.2 Practices and Guidelines Regarding Lactating Mothers' Intake of Medications, Alcohol and Caffeine

Practices regarding maternal intake of medications and allowance of concurrent feeding of MOM to the infant are presented in Table 3. Additional medications that were specified in the open‐ended question which prohibited MOM feeding included radioactive substances, high‐dose hormones, cytostatic agents and lithium.

**TABLE 3:** Maternal and donor medications.

Pharmaceuticals	MOM					DHM		
	*N* = 24					*N* = 24		
	Immediate consumption allowed	Immediate consumption not allowed (MOM frozen and used later)	Discarded	Both/either or	Missing	Allowed/permitted	Not allowed/permitted	Missing
Hormone preparations (e.g., thyroid hormone and insulin)	21	21			3	19	1	4
Local treatment with inhalation preparations for asthma	22	22			2	16	4	4
Local treatment of skin, nose and eyes (e.g., corticosteroids and sodium cromoglicate [Lomudal])	22	22			2	17	3	4
Contraceptives in the form of progestogens	20	18			4	13	6	5
Pain‐relieving medications: temporary treatment, e.g., after a caesarean section (< 7 consecutive days)	22	17	3	1	2	NA	NA	NA
Pain‐relieving medications: long‐term treatment (> 7 consecutive days)	21	9	5	2	3	NA	NA	NA
Pain‐relieving medications	NA	NA	NA	NA	NA	8	12	4
Antihypertensives	22	14	2	2	2	3	17	4
Psychopharmaceuticals	22	7	3	2	2	2	18	4
Herbal medicines	19	13	1		5	2	16	6

Nine out of 24 responding units reported having guidelines regarding maternal alcohol consumption. Responses to the open‐ended question regarding alcohol consumption included: ‘At least two hours after a small glass of wine’ and ‘There is no guideline for this; however, if a mother were to ask about it, we would explain that knowledge regarding alcohol consumption during breastfeeding is very limited, and we do not know how it affects the infant. We refer to the Swedish Food Agency's guidelines, which state that it is likely not harmful to the infant if the mother consumes 1–2 glasses of wine per week. Mothers of severely ill infants cared for in the neonatal unit rarely ask about alcohol consumption’. Others responded that a delay in pumping/breastfeeding was needed for 2, 12 or 24 h. Three out of 23 units had guidelines regarding maternal coffee and caffeine intake. Responses to the open‐ended question regarding coffee and caffeine consumption included: ‘a reasonable amount, three cups daily’ and ‘one can eat/drink everything in moderation’.

### 3.4 Routines Regarding Donor Human Milk

The criteria used for determining infant eligibility of DHM feeding varied (Table 4). Most units provided DHM to infants born before 34–35 weeks' gestation and to all infants who had undergone abdominal surgery. Some units also offered DHM to infants with hypoglycaemia, growth restriction or those undergoing hypothermia treatment after asphyxia independent of gestational age at birth. Responses to the open‐ended question included: ‘It depends a bit on availability, but for most of the year we can offer it to all infants’ and ‘If there is a large supply, it is also offered to full‐term infants with issues such as gastrointestinal problems’. Moreover, several units had a clear prioritisation system regarding which infants should receive the DHM with the highest protein content, with the lowest gestational age and weight being given the highest priority. If there was an abundant supply of DHM, it could also be offered to infants in the paediatric intensive care unit or maternity ward when needed, but infants cared for in the neonatal units were always prioritised.

### 3.5 Storage, Nutritional Analysis and Fortification of Human Milk

Information regarding current practices of storage, as well as nutritional analysis and fortification of human milk (both MOM and DHM) in Swedish neonatal units is presented in Table 4.

**TABLE 4:** Milk handling and procedures at the Neonatal unit.

	MOM		DHM
	*N* = 24		*N* = 24
	*n*/(missing)		*n*/(missing)
**Storage**			
**Time for MOM in room temperature**	**Freshly expressed MOM**	**Time for DHM in room temperature**	**Thawed pasteurised DHM**
Immediately to refrigerator	5	Directly from fridge at time for feeding	12
1 h	7	Should be given within 20–30 min	2
2 h	2	Should be given within 1 h	3
4 h	7	Should be given within 2 h	3
6 h	3	According to Milknet guidelines	1
		1 h in room, 3 h for thawing	1
**Time in refrigerator**		**Time in refrigerator**	
24 h	2	24 h	6 (1)
48 h	18	48 h	16 (1)
72 h	4	72 h	1 (1)
**Time in freezer** *		**Time in freezer** *	
3 months (−18°C)	3^a^	3 months (−18°C)	3 (1)*
6 months (−21°C)	22	6 months (−21°C)	21 (1)
**Nutrition analysis** *			
**Analyses performed at unit/hospital**	**MOM** **24**	**Analyses performed at unit/hospital**	**DHM** **23 (1)**
Yes	14	Yes	14
Sometimes	2	Sometimes	1
No, performed at HMB at other hospital	8	No, performed at HMB at other hospital	8
**Criteria for nutritional analysis of MOM**	**24**	**Criteria for infant eligibility of DHM feeding**	**24**
Gestational age and/or birth weight (not specified)	4	Only infants born preterm or SGA/IUGR	12^b^
All infants < 32 weeks gestation	7	Infants at units outside neonatal care	11^c^
Birth weight below 1500 g	2	Other	8^d^
Infants < 34–35 weeks gestation	9		
Hospital stays long enough (not specified)	1		
Hospital stay > 7 days	1		
**How often is nutrition analysis of MOM performed?**	**MOM** **24**	**Prioritisation of DHM with higher protein content**	**DHM** **24**
Regularly	22	Yes	17^e^
Occasionally	2	No	7
Once a week	14		
Every other week	5		
Other, individual depending on situation and over time	3		
**Procedure for milk collection for nutritional analysis**	**23/24 (1)**		
A mixed sample of collected milk over a 24‐h period	21		
A mixed sample of pumped milk at 4–5 pumping's	2		
**Minimum amount of MOM to perform nutritional analysis**	**16/24 (8)**		
3 mL	4		
8 mL	2		
10 mL	7		
12 mL	1		

* Analyse for its nutritional content (i.e., energy, protein, fat and carbohydrates).

^a^ One unit given both answers.

^b^ Free text answers were gestational week 34 (*n* = 3), gestational week 35 (*n* = 2), mainly to preterm infants and SGA but if enough supply also to term infants with gastroenteral problems, infants in need of gastroenteral surgery, infant weight < 1500 g, < 2000 g (*n* = 2), < 2500 g and not mature enough to have infant formula, infants with hypoglycaemia, and according to supply but mainly all infants.

^c^ Free text answers were, postnatal care (*n* = 9), delivery ward (*n* = 3), paediatric ward or in homecare in specific cases to infants with gastrointestinal problems, in exceptional cases due to medical reasons, and infants on leave from ward to home.

^d^ Free text answers were, as many as possible when enough supply but always infants < 35 weeks and infants with hypoglycaemia, all infants < 34 gestational weeks and > 34 weeks if infant formula is not tolerated, all infants < 32 gestational weeks and if enough supply to all infants instead of infant formula with an aim to give as first feed if the mother cannot express her own milk, at few occasions also to term infants with problems of vomiting, all preterm infants are prioritised but sometimes term infants with difficulties feeding and/or hypoglycaemia, infants to mothers with insulin treated diabetes, and one unit did not handle donor milk at their unit.

^e^ Free text answers were that infants with the lowest gestational age and infant weight are prioritised (*n* = 13), based on three categories according to protein content, and in consultation with dietitian and staff with a focus on nutrition.

### 3.6 Human Milk Donations and Processing of Donor Human Milk

All responding units participated, to various degrees, in the processing of DHM (Table 1). Depending on the degree of the handling process, the maximum answers on part 1 of the survey (pasteurisation process excluded) were 24, and in part 2 (pasteurisation process included) the maximum was 19. The number of staff involved in handling human milk donations varied across neonatal units, between one to five members of the staff to all members of the staff (Table 5). The professions responsible for handling DHM were assistant nurses, or both assistant and registered nurses. One unit relied solely on registered nurses for handling DHM, and in one ward, assistants (with no formal education) managed this process. In 20 units, the staff handling DHM also worked with patient care. Two units reported having separate staff dedicated exclusively to handling milk donations. In those units with staff involved in both tasks, 11 units reported that staff handled both responsibilities during the same shift, while nine units assigned different tasks, DHM handling or infant care, on separate days.

**TABLE 5:** Handling processes for donor human milk and routines at the HMB.

Handling processes for donor human milk *EXCEPT* pasteurisation	
Definition: The handling process of DHM involves for example contact with human milk donors, collecting donor health declarations and performing serological screening of donors, providing donors with breast milk pumps and collecting milk donations	
	*n*/*N* (missing)
Staff working with handling processes	
Professions working with handling DHM	24/24
Assistant nurses (college‐level education in nursing)	16
Childcare assistants (college‐level education in Children and Leisure studies)	11
Registered nurses	6
Ward assistants (with no formal medical/nursing education)	1
Do the staff work both with handling DHM and with patient care?	22/24 (2)
Yes	20
No	2
Do the staff work with handling DHM and with patient care on the same day?	18/20 (2)
Yes	11
No	9
Routines regarding recruitment of milk donors	
Recruitment of milk donors	23/24 (1)
At the neonatal unit	22
Postnatal ward	19
Chid Health Centres	18
Social media platforms	18
Postnatal follow up visits	11
Maternity clinics	10
Posters and information pamphlets	10
Transportation of donor milk to HMB	24
By donors	22
By home care staff	7
Other alternatives	7
Serological screening at donor recruitment	22/24 (2)
HIV 1/2	22
HTLV‐I	20
HTLV‐II	19
Hepatitis B	22
Hepatitis C	21
Syphilis	4
Time limits for milk donations	23/24 (1)
3 months	12
4 months	1
6 months	2
3 months + 3 months	7
Based on nutritional analysis	1
Are new serological screening required after 3 months, if the donor wish to continue donating?	22/24 (2)
Yes	20
No	2
Have you needed to decline donors related to organisational shortages?	20/24 (4)
Yes	9
No	11

Routines in the HMB *INCLUDING* pasteurisation	
Definition: The pasteurisation process of DHM involves pasteurising milk donations and performing bacteriological testing and nutritional analysis of the DHM	
	*n*/*N* (missing)
Staff working in the HMB	
How many of the staff works with pasteurisation processes	19/19
1 person	1
1–2 persons	5
3 persons	5
4 persons	5
5 persons	1
All staff in the unit	2
Professions working in the HMB	17/18 (1)
Assistant nurses (college‐level education in nursing)	15
Childcare assistants (college‐level education in Children and Leisure studies)	11
Registered nurses	6
Ward assistants (with no formal medical/nursing education)	1
Do the staff work both in the HMB and with patient care?	17/18 (1)
Yes	14
No	3
Do the staff work in the HMB and with patient care on the same day?	14/14 (0)
Yes	3
No	11
Are all donor milk pasteurised?	19
Yes	18
No, if donor milk is too old it is discarded	1
Do you mix milk from different milk donors (pooling)?	19
Yes	0
No	18
Sometimes	1
When is nutrition analysis of DHM performed?	13/19 (6)
Before pasteurisation	11
After pasteurisation	1
Both before and after pasteurisation	1
Protein analysed	
True	12 (2)
Crude	4 (2)
Do not know	6 (2)
Is the donated breast milk evaluated for bacterial content according to the following guidelines?	18
No potentially pathogenic bacteria such as beta‐haemolytic streptococci group A, C or G, streptococci group B, Listeria or Salmonella	17 (1)
< 107 cfu/L of *Staphylococcus aureus*	16 (2)
< 107 cfu/L of Enterobacteriaceae	16 (2)
< 107 cfu/L of *Pseudomonas aeruginosa* or other Pseudomonas species	16 (2)
< 107 cfu/L of *Stenotrophomonas maltophilia*	15 (3)
< 107 cfu/L of Acinetobacter	16 (2)
Aerobic bacteria, e.g., coagulase‐negative staphylococci or alpha‐streptococci	15 (3)
When is the bacterial testing conducted?	18
Before pasteurisation	17
Before and after pasteurisation	1
How is the bacterial testing conducted?	19
From liquid milk, fresh or thawed	10
From frozen milk, a scratch‐test	5
Both methods	4
Is all donor milk tested for bacterial growth?	18/19 (1)
A pooled sample from each batch	9
A random sample from one batch	4
The oldest milk	2
At recruitment, then occasionally	1
Do you provide other units or hospitals with DHM?	18/19 (1)^a^
Yes, other units in our hospital	9
Yes, other hospitals within our region	6
Yes, other hospitals outside our region	3
No, we do not provide DHM to other units/hospitals	5
If excess of DHM, do you share with other hoppitals or regions?	19
Yes	5
No	5
Sometimes	9

^a^ Free text answers were, postnatal ward (*n* = 5), delivery ward (*n* = 2), other neonatal units.

#### 3.6.1 Recruitment of Human Milk Donors

The primary recruitment of new donors occurred at the neonatal units, where all units reported recruiting new donors. Additional recruitment sites included the postnatal ward, Child Health Centres, social media platforms, postnatal follow‐up visits, maternity clinics, and posters and informational pamphlets distributed in various healthcare settings (Table 5). When DHM is scarce, advertising efforts are extended to newspapers, radio and social media platforms. All units provided milk donors with a free electric breast pump. In addition, all units offered the donor financial compensation, between SEK 125 per litre and SEK 250 per litre (SEK 125–250 per litre currently corresponds to USD 11–23). Five units reported that they never needed to decline any milk donors. Other units mentioned declining donors on isolated occasions, up to turning away donors 10–20 times per year. Reasons for not accepting donors included bacteriological testing indicating milk not reaching the standard, excess donors, limited storage capacity, limited supply of breast pumps, donor medical treatments, donors not meeting the health declaration requirements and time limits of 3 months after birth for donation. Regarding transportation of breast milk to the milk bank, most DHM was delivered by the donors themselves. Other methods included transport by home care staff or alternative transport arrangements (Table 5), such as donors leaving frozen breast milk at the nearest Health Care Centre or Child Health Clinic, from which it would be transported frozen by the ordinary lab transport vehicle used for transport of other samples and material in the region. In one region, nutrition assistants from the neonatal unit collected milk from mothers in their homes once a week. Sixteen of the units had a time limit on how long a woman could donate breast milk, whereas seven did not. The time limit for donations ranged from 3 months post‐birth, where some allowed an extension of the donation period by an additional 3 months. One unit reported a time limit of 4 months, and two units had a limit of 6 months. One unit did not impose a specific time limit; instead, the decision was based on the results of breast milk nutritional analysis.

#### 3.6.2 Practices and Guidelines Regarding Donor Health and Intake of Medications, Alcohol, Caffeine and a Vegan Diet

Most units reported having local routines for donor health declarations, based on the national guidelines. Responses to the open‐ended question regarding medical treatments for human milk donors indicated that the neonatal units were very restrictive in permitting donors undergoing medical treatment. One unit noted that their criteria for permitted medications were stricter than those for breastfeeding one's own infant; donors may only take medications approved by the guidelines, and any donation that did not comply was declined. The physicians used the website Janusinfo [22] to assess the safety of medications on an individual basis. Some analgesics, such as paracetamol, ibuprofen and non‐steroidal anti‐inflammatory drugs (NSAID), were approved for short‐term use (Table 3). Approval of other medications depended on the dosage. Most antiepileptics, neuroleptics, opioids, immunomodulating drugs and thyreostatics were not permitted for human milk donors. Twenty‐one of 24 units answered questions regarding whether alcohol consumption or a vegan diet was permitted for donors; three were missing. Seven out of 21 neonatal units accepted some level of alcohol consumption among milk donors, while 14 units did not. For those units permitting alcohol consumption, it was reported that occasional intake was acceptable, with a maximum of one glass of wine per week. Moreover, responses revealed that it was recommended that donors refrained from expressing milk for donation for 2–24 h after alcohol consumption. Similarly, seven out of 21 units had guidelines regarding caffeine intake, whereas 14 units did not. The reported guidelines indicated that a reasonable caffeine intake was a maximum of three cups of coffee per day. Moreover, one unit also noted that donors should avoid energy drinks due to their high caffeine content. Fourteen units permitted donors adhering to a vegan diet, while seven units did not. Two units reported evaluating vegan diets of donors to determine if nutritional supplementation was necessary; in one unit this was performed by a dietician and the other unit by a physician.

#### 3.6.3 Pasteurising and Pooling of Donor Milk

Nineteen units pasteurised DHM (Table 1), and the pasteurisation method used in all units was Holder pasteurisation. Regarding pooling of DHM, 18 of the 19 pasteurising units reported not practising pooling whereas one unit occasionally pooled milk from different donors when there was a shortage of DHM.

#### 3.6.4 Serological Screening of Potential Milk Donors

The serological screening at recruitment of donors was mostly uniform among respondents (Table 5). One unit did not perform Hepatitis C and Syphilis at the time of recruitment, noting that these tests had already been conducted during pregnancy. If a woman had donated milk for 3 months, 20 units indicated that new serological screening should be conducted if she wished to continue donating. In contrast, two units responded that they did not require an additional serological screening for prolonged milk donation, and three units reported that they did not accept milk donations after 3 months.

#### 3.6.5 Bacteriological Testing of Donor Milk

Apart from those bacteria presented in Table 5 the survey responses to the open‐ended question indicated that growth of the following bacteria in DHM was not accepted: Candida, Serratia, extended‐spectrum beta lactamase (ESBL)‐producing bacteria, methicillin‐resistant *Staphylococcus aureus* (MRSA) and *Bacillus cereus* . Furthermore, one neonatal unit reported that for the total number of aerobic bacteria, including coagulase‐negative staphylococci or alpha‐streptococci, there was no upper limit. Another unit reported that for mould or bacilli, they looked at the donor's history: if it was the first occasion, they re‐cultivated the sample and if it still showed presence of mould or bacilli, they requested a new sample of the same milk. If the new sample continued to be positive, it was rejected.

#### 3.6.6 Sharing of DHM Within Swedish Healthcare

The provision of pasteurised DHM to other units primarily occurred within the hospital, for example between the birth unit and the neonatal unit, and within the healthcare region. It was less common for DHM to be provided to other healthcare regions (Table 5). In cases of excess DHM, six units donated or sold milk to other hospitals and regions, while five units did not. The price set for DHM varied between USD 22–88 per litre. One unit reported no fixed price and stated that the price would be negotiated at the time of sale. The varying costs may depend on whether the DHM was pasteurised, with a higher price for pasteurised DHM.

## 4 Discussion

This study highlights variations in practices for handling both MOM and DHM in Swedish neonatal units and HMBs, suggesting a need for improved implementation, clarification and the updating of national guidelines.

### 4.1 Supporting Mothers to Provide MOM as the First Choice

All units reported giving lactation support to mothers, but only 11 reported having written guidelines. According to the WHO/UNICEF's 10 Steps to Successful Breastfeeding, all facilities providing maternity and newborn services should have written guidelines which are routinely communicated to staff and parents [23].

Only a third of the units in this study reported giving information about the differences between MOM and DHM. Furthermore, there were differences between units regarding staff training and education on lactation and breastfeeding, ranging from no training at all to giving all new staff education and regular training once a year. These differences may lead to families meeting staff with different competencies, which may affect their lactation and breastfeeding process [24, 25]. However, more than half of the units used colostrum kits when informing parents. The colostrum kit was developed in a previous quality improvement project to help staff give standardised basic information and tools for milk collection to parents regarding milk expression and production, aiming to empower parents with knowledge about the physiology of initiation of a milk production. Hellström et al. have demonstrated reduced time to first infant colostrum intake after implementing the colostrum‐kit in the NICU [18]. They further demonstrated that previous differences in time to infant colostrum intake related to mode of delivery were reduced and that the effect of the implementation persisted over time [18].

In a review, Meier et al., the authors describe the evidence, mechanism and priorities for research and practice regarding DHM, demonstrating the superiority of MOM for the reduction of mortalities and to be cost effective [6]. They conclude that providers of neonatal care need to find ways to support the superiority of MOM over DHM that results in higher doses and longer periods of MOM intake for infants [6].

### 4.2 Handling, Storage and Fortification of Human Milk

There were differences among units regarding whether MOM was given frozen in chronological order or mainly given fresh. There are ongoing studies on whether giving fresh MOM may improve infant health outcomes [26, 27]. Further research on the potential biological benefits of fresh MOM and the changes that occur during storage is needed [28].

Furthermore, there was variation in fortification routines. Fortification is standard practise for very‐low‐birth‐weight (VLBW) infants and is needed to support growth and development, but there may be dilemmas regarding the type of fortification, when timely initiation and continuation of fortification, and the duration of fortification [29]. In a study by Ericson et al., they found no significant differences in neurological outcomes, growth or health in children aged 6 years between moderately preterm infants (32–36 weeks of gestation) who were given fortification or formula compared with those exclusively given breast milk [30]. Additionally, in a review by Hard et al., the authors demonstrate that donor milk does not promote growth and development as well as MOM [7], why infants receiving donor milk may have an increased need of fortification. Moreover, there were differences in routines regarding the preparation of fortification, ranging from at bedside for every feeding to once every 24 h. Fortification has been associated with an increase of osmolality in human milk, which may happen immediately after fortification [31]. Furthermore, concerns have also been raised regarding microbial growth in fortified DHM, which may be affected by storage time in a refrigerator [32].

### 4.3 Acceptance of Medications, Caffeine and Alcohol

There is a lack of guidelines for maternal medication related to infant MOM intake in Sweden. Several discrepancies were observed among the neonatal units regarding the acceptance of maternal medication intake and providing MOM to their infants. These differences were mainly related to analgesics, contraceptives containing progesterone, antihypertensives and psychopharmaceuticals. These differences indicate a need for clarified and updated guidelines regarding what medications should be considered safe to consume by a mother currently providing MOM to her infant.

In contrast, stricter guidelines regarding medical treatment were reported for milk donors. However, there were differences between HMBs, most commonly regarding analgesics, antihypertensives and psychopharmaceuticals. Again, this suggests a need for clarifications and updated evidence in future Milknet guidelines. There were also differences among units regarding both MOM and DHM, as to whether they had guidelines for alcohol intake, caffeine intake and vegan diets. The results indicate a need for clearer guidelines in these areas.

### 4.4 Routines for Milk Donations

#### 4.4.1 Milk Donations

Only five units reported never having declined milk donations, while other units reported a frequency of occasionally up to 20 times a year. Several of the reasons mentioned for rejecting donors were modifiable, such as inability to take on willing donors, limited storage capacity and breast pumps, as well as time limits on donation. If the storage capacity were improved in Swedish HMBs, donor milk could be used throughout the infants' hospital stay at both the neonatal and postnatal units. In most units today, DHM is only given to preterm infants born before 34 weeks' gestation, being phased over to formula when the infants reach 34 weeks' gestation if MOM is unavailable or does not meet the infants' needs. In both the WHO/UNICEF global guidelines [23] and the Swedish guidelines [20], DHM is recommended as the next choice if MOM is unavailable or does not meet the infant's needs.

There were also reported differences in where the milk donors could deliver their milk. Most units reported that the donors had to leave the milk at the milk bank, but other units allowed mothers to deliver their milk to other health services closer to their homes, where it could be transported by regular channels (e.g., as used for blood samples) within the health and welfare organisation. Other units reported collecting DHM at the milk donors' homes. It is imperative to establish a system that facilitates the donation of milk for the donors. This is particularly relevant given that donors are often women who have recently given birth and may face difficulties in travelling to HMBs to deliver their milk [33, 34]. To support and facilitate milk donations, the above examples may be implemented by others.

#### 4.4.2 Serological Screening of Milk Donors

All units performed serological screening when recruiting new milk donors. Most units that accepted donor milk for more than 3 months reported repeating the initial serological screening for a new donation period. Enabling repeated serological screening may facilitate a longer period of milk donation and improve the availability of DHM, extending its use to all infants needing supplementation during their hospital stay.

#### 4.4.3 Bacteriological Testing of Donor Milk

Most HMBs reported bacteriological testing before pasteurisation, with only one unit stating that testing is done both before and after pasteurisation. Routines for bacterial testing before pasteurisation have been discussed internationally, as related to milk not being accepted for donation, even though pathogens will be eliminated through pasteurisation [35], which may lead to a lack of supply of donor milk. Both the American Academy of Paediatrics (AAP) [36] and the Human Milk Banking Association of North America (HMBANA) guidelines (2024) [37] recommend bacteriological testing of each batch after pasteurisation. Testing after pasteurisation may be beneficial for both the safety and increased availability of donor milk.

There were also different routines reported for bacteriological testing of the same batch of donated milk, related to testing all milk in a batch or only random samples of the milk.

Most units reported accepting donor milk according to the Milknet guidelines [20]. However, some units reported evaluating and imposing restrictions based on other possible pathogens as well, such as Candida, Serratia, ESBL, MRSA and *Bacillus cereus* . These results and a review of new literature and evidence indicate a need for an update of the revised Milknet guidelines for possible pathogens to be screened.

#### 4.4.4 Handling and Pasteurisation

All units reported pasteurising all donor milk. Guidelines related to handling and pasteurisation vary among countries [38], but there is consensus on the need for quality control to ensure safe donor milk [38]. Since the pasteurisation process affects many of the bioactive components of human milk, both pasteurisation methods and the possibility of not pasteurising donor milk have been subjects of research and discussion [38].

#### 4.4.5 Collaboration to Utilise Resources When There Is Excess Donor Milk

In Sweden there is no national HMB organisation. Collaboration among HMBs differed in terms of the provision of pasteurised donor milk to other units in cases of excess. The establishment of a network to provide other units with donor milk is imperative to enhance collaboration in Sweden, preventing the discarding of surplus donor milk in one unit when supply is inadequate in another. An organization of Swedish HMBs on a national level could facilitate collaboration and optimise the use of DHM.

### 4.5 The Importance of Supporting Both MOM and DHM Supply: Ways Forward?

There are several factors beyond pasteurisation that may affect the clinical properties of DHM. Meier et al. [6] highlighted that factors including mammary gland maturity, lactation stage, freeze–thaw cycles, and the storage and handling of DHM. They further emphasised a specific mismatch that occurs when MOM is replaced with DHM in the early postnatal period. This is related to previous studies suggesting that the composition of MOM during the early stage of lactation reflects the immaturity of the mammary gland and is uniquely tailored to the infant's biological needs during this critical window after birth [6].

MOM contains numerous bioactive factors, including microbiota that are involved in the health effect for infants [8]. Since DHM is pasteurised, several bioactive factors and microbes are reduced or abolished [7]. There have been studies on possibilities to recolonize donor milk with microbiota from MOM, a milk microbiome transplantation [39, 40, 41]. This approach is a way to personalise DHM with the individual mother's milk microbiota [39].

Furthermore, in a study by Tan et al., the authors demonstrated that very preterm infants fed MOM, compared to infants fed DHM, had a trajectory of immune development with greater resemblance to term infants [42]. This normalisation in development was explained by NK cell development and not by differences in microbial colonisation, suggesting a direct effect of bioactive factors in milk. These results strengthen the need to continue to support maternal lactation in neonatal care.

Hendricks‐Munoz et al. [43] suggested that utilising DHM while supporting MOM at discharge can reduce the need for formula in VLBW infants. However, they emphasised that without additional resources for lactation support, increasing MOM intake at discharge may be challenging. They advocated individualised maternal education and lactation support to positively influence MOM feeding practices at discharge [43]. Further, Kontopodi et al. [44] conducted a web‐based survey of European HMBs, revealing significant variability in practices across the continent. This variability highlights opportunities to enhance the effectiveness of DHM banking. Despite well‐established national guidelines in Sweden, the present study also found considerable variations in practices, underscoring the need for greater standardisation.

### 4.6 Method Discussion

The survey contained 102 questions, some similar in content but related to different aspects of human milk handling, which may have affected the response rate of 63% for part 1 of the survey and 68% for part 2. Several reminders were sent to improve the response rate. Furthermore, in the information provided with the survey, different professions were asked to answer different questions, which also may have negatively affected the response rate. However, receiving answers from different professions may have strengthened the results within this field. Additionally, having responses from neonatal units of different care levels in Sweden, as well as responses from all regions in the country, may have strengthened the generalizability of the results nationally.

## Author Contributions

**Ylva Thernström Blomqvist:** conceptualization, investigation, writing – original draft, methodology, validation, visualization, writing – review and editing, project administration, formal analysis, data curation, supervision. **Anna‐My Lund:** conceptualization, investigation, writing – original draft, methodology, validation, visualization, writing – review and editing, formal analysis, data curation. **Fredrik Ahlsson:** conceptualization, methodology, writing – review and editing. **Jenny Ericson:** conceptulization, methodology, writing – review and editing. **Charlotta Karlsson:** conceptulization, methodology, writing – review and editing. **Josefin Lundström:** conceptulization, methodology, writing – review and editing. **Ingela Sandström:** conceptulization, methodology, writing – review and editing. **Anna Gustafsson:** conceptualization, investigation, writing – original draft, methodology, validation, visualization, writing – review and editing, formal analysis, project administration, data curation, supervision.

## Funding

The authors have nothing to report.

## Conflicts of Interest

The authors declare no conflicts of interest.

## Supporting information


**Appendix S1:** apa70448‐sup‐0001‐AppendixS1.docx.

**Appendix S2:** apa70448‐sup‐0002‐AppendixS2.docx.

## Data Availability

The data that support the findings of this study are available on request from the corresponding author. The data are not publicly available due to privacy or ethical restrictions.

## References

[1] C. G. Victora , R. Bahl , A. J. Barros , et al., “Breastfeeding in the 21st Century: Epidemiology, Mechanisms, and Lifelong Effect,” Lancet 387 (2016): 475–490. 26869575 10.1016/S0140-6736(15)01024-7

[2] E. M. Nagel , K. M. Elgersma , T. T. Gallagher , K. E. Johnson , E. Demerath , and C. A. Gale , “Importance of Human Milk for Infants in the Clinical Setting: Updates and Mechanistic Links,” Nutrition in Clinical Practice 38, no. Suppl 2 (2023): S39–S55. 37721461 10.1002/ncp.11037 PMC10513735

[3] N. D. Embleton , S. Jennifer Moltu , A. Lapillonne , et al., “Enteral Nutrition in Preterm Infants (2022): A Position Paper From the ESPGHAN Committee on Nutrition and Invited Experts,” Journal of Pediatric Gastroenterology and Nutrition 76 (2023): 248–268. 36705703 10.1097/MPG.0000000000003642

[4] P. P. Meier , T. J. Johnson , A. L. Patel , and B. Rossman , “Evidence‐Based Methods That Promote Human Milk Feeding of Preterm Infants: An Expert Review,” Clinics in Perinatology 44 (2017): 1–22. 28159199 10.1016/j.clp.2016.11.005 PMC5328421

[5] L. A. Parker , R. Koernere , K. Fordham , et al., “Mother's Own Milk Versus Donor Human Milk: What's the Difference?,” Critical Care Nursing Clinics of North America 36 (2024): 119–133. 38296370 10.1016/j.cnc.2023.09.002

[6] P. Meier , A. Patel , and A. Esquerra‐Zwiers , “Donor Human Milk Update: Evidence, Mechanisms, and Priorities for Research and Practice,” Journal of Pediatrics 180 (2017): 15–21. 27773337 10.1016/j.jpeds.2016.09.027 PMC5183469

[7] A. L. Hard , A. K. Nilsson , A. M. Lund , I. Hansen‐Pupp , L. E. H. Smith , and A. Hellstrom , “Review Shows That Donor Milk Does Not Promote the Growth and Development of Preterm Infants as Well as Maternal Milk,” Acta Paediatrica 108 (2019): 998–1007. 30565323 10.1111/apa.14702 PMC6520191

[8] O. Ballard and A. L. Morrow , “Human Milk Composition: Nutrients and Bioactive Factors,” Pediatric Clinics of North America 60 (2013): 49–74. 23178060 10.1016/j.pcl.2012.10.002 PMC3586783

[9] L. A. Hanson , “Session 1: Feeding and Infant Development Breast‐Feeding and Immune Function,” Proceedings of the Nutrition Society 66 (2007): 384–396. 17637091 10.1017/S0029665107005654

[10] J. Neu , “Preterm Infant Nutrition, Gut Bacteria, and Necrotizing Enterocolitis,” Current Opinion in Clinical Nutrition and Metabolic Care 18 (2015): 285–288. 25807349 10.1097/MCO.0000000000000169 PMC4417625

[11] B. E. Lechner and B. R. Vohr , “Neurodevelopmental Outcomes of Preterm Infants Fed Human Milk: A Systematic Review,” Clinics in Perinatology 44 (2017): 69–83. 28159210 10.1016/j.clp.2016.11.004

[12] B. M. Henrick , X. D. Yao , L. Nasser , A. Roozrogousheh , and K. L. Rosenthal , “Breastfeeding Behaviors and the Innate Immune System of Human Milk: Working Together to Protect Infants Against Inflammation, HIV‐1, and Other Infections,” Frontiers in Immunology 8 (2017): 1631. 29238342 10.3389/fimmu.2017.01631 PMC5712557

[13] J. Cortez , K. Makker , D. F. Kraemer , J. Neu , R. Sharma , and M. L. Hudak , “Maternal Milk Feedings Reduce Sepsis, Necrotizing Enterocolitis and Improve Outcomes of Premature Infants,” Journal of Perinatology 38 (2018): 71–74. 29048409 10.1038/jp.2017.149

[14] A. J. Lewandowski , P. Lamata , J. M. Francis , et al., “Breast Milk Consumption in Preterm Neonates and Cardiac Shape in Adulthood,” Pediatrics 138 (2016): e20160050. 27302980 10.1542/peds.2016-0050 PMC6198929

[15] A. El‐Khuffash , A. J. Lewandowski , A. Jain , A. Hamvas , G. K. Singh , and P. T. Levy , “Cardiac Performance in the First Year of Age Among Preterm Infants Fed Maternal Breast Milk,” JAMA Network Open 4 (2021): e2121206. 34448867 10.1001/jamanetworkopen.2021.21206 PMC8397926

[16] K. Miliku , T. J. Moraes , A. B. Becker , et al., “Breastfeeding in the First Days of Life Is Associated With Lower Blood Pressure at 3 Years of Age,” Journal of the American Heart Association 10 (2021): e019067. 34284597 10.1161/JAHA.120.019067 PMC8475685

[17] I. Levene , M. A. Quigley , M. Fewtrell , and F. O'Brien , “Does Extremely Early Expression of Colostrum After Very Preterm Birth Improve Mother's Own Milk Quantity? A Cohort Study,” Archives of Disease in Childhood. Fetal and Neonatal Edition 109 (2024): 475–480. 38442953 10.1136/archdischild-2023-326784 PMC11347236

[18] S. Hellström , K. Linden , V. Sengpiel , and A. Elfvin , “Implementing a Colostrum‐Kit Reduces the Time to First Colostrum for Neonates Admitted to the NICU ‐ a Retrospective Observational Study,” International Breastfeeding Journal 19 (2024): 77. 39548520 10.1186/s13006-024-00682-5 PMC11566270

[19] Y. Thernstrom Blomqvist , J. Agren , and V. Karlsson , “The Swedish Approach to Nurturing Extremely Preterm Infants and Their Families: A Nursing Perspective,” Seminars in Perinatology 46 (2022): 151542. 34911652 10.1016/j.semperi.2021.151542

[20] S. Polberger , “Guidelines for the Use of Human Milk and Milk Handling in Sweden, Milknet, Version 3.0,” (2016), https://www.milknet.se/_files/ugd/f2fee3_e1089e304b5a44fa994f137bf094b3cc.pdf2016.

[21] U. H. Graneheim , B. M. Lindgren , and B. Lundman , “Methodological Challenges in Qualitative Content Analysis: A Discussion Paper,” Nurse Education Today 56 (2017): 29–34. 28651100 10.1016/j.nedt.2017.06.002

[22] “Janusinfo,” (2024), https://janusinfo.se/.

[23] World Health Organization and the United Nations Children's Fund (UNICEF) , “Protecting, Promoting and Supporting Breastfeeding in Facilities Providing Maternity and Newborn Services: Implementing the Revised Baby‐Friendly Hospital Initiative 2018,” (2024), https://iris.who.int/bitstream/handle/10665/272943/9789241513807‐eng.pdf?sequence=192018.

[24] A. M. Rohini , S. Elavally , and G. Saradakutty , “Effectiveness of Breastfeeding Education Compared to Standard Hospital Information on Exclusive Breastfeeding Among Mothers: A Systematic Review,” Journal of Education Health Promotion 11 (2022): 125. 35677266 10.4103/jehp.jehp_708_21 PMC9170204

[25] A. Gavine , S. MacGillivray , M. J. Renfrew , L. Siebelt , H. Haggi , and A. McFadden , “Education and Training of Healthcare Staff in the Knowledge, Attitudes and Skills Needed to Work Effectively With Breastfeeding Women: A Systematic Review,” International Breastfeeding Journal 12 (2016): 6. 28167998 10.1186/s13006-016-0097-2 PMC5288894

[26] H. Sun , Y. Cao , S. Han , et al., “A Randomized Controlled Trial Protocol Comparing the Feeds of Fresh Versus Frozen Mother's Own Milk for Preterm Infants in the NICU,” Trials 21 (2020): 170. 32046760 10.1186/s13063-019-3981-4 PMC7014600

[27] H. Sun , S. Han , R. Cheng , M. Hei , F. Kakulas , and S. K. Lee , “Testing the Feasibility and Safety of Feeding Preterm Infants Fresh Mother's Own Milk in the NICU: A Pilot Study,” Scientific Reports 9 (2019): 941. 30700726 10.1038/s41598-018-37111-7 PMC6353969

[28] C. E. Briere and J. Gomez , “Fresh Parent's Own Milk for Preterm Infants: Barriers and Future Opportunities,” Nutrients 16 (2024): 362. 38337647 10.3390/nu16030362 PMC10857054

[29] A. B. Hair , B. Scottoline , and M. Good , “Dilemmas in Human Milk Fortification,” Journal of Perinatology 43 (2023): 103–107. 36097287 10.1038/s41372-022-01502-6 PMC10317058

[30] J. Ericson , F. Ahlsson , D. Wackernagel , and E. Wilson , “Equally Good Neurological, Growth, and Health Outcomes up to 6 Years of Age in Moderately Preterm Infants Who Received Exclusive vs. Fortified Breast Milk—A Longitudinal Cohort Study,” Nutrients 15 (2023): 15. 10.3390/nu15102318 PMC10223744 37242201

[31] N. Kreins , R. Buffin , D. Michel‐Molnar , V. Chambon , P. Pradat , and J. C. Picaud , “Individualized Fortification Influences the Osmolality of Human Milk,” Frontiers in Pediatrics 6 (2018): 322. 30430102 10.3389/fped.2018.00322 PMC6220443

[32] C. Mandru , M. T. Perrin , R. Iyer , et al., “Bacterial Content of Fortified and Unfortified Holder Pasteurized Donor Human Milk During Prolonged Refrigerated Storage,” Journal of Pediatric Gastroenterology and Nutrition 69 (2019): 487–492. 31232828 10.1097/MPG.0000000000002427

[33] E. Olsson , B. Diderholm , and Y. T. Blomqvist , “‘Paying It Forward’—Swedish Women's Experiences of Donating Human Milk,” Journal of Human Lactation 37 (2021): 87–94. 33275499 10.1177/0890334420979245 PMC7907995

[34] Y. T. Blomqvist and E. Olsson , “Experiences of Breast Milk Donors in Sweden: Balancing the Motivation to Do Something Good With Overcoming the Challenges It Entails,” International Breastfeeding Journal 19 (2024): 60. 39217315 10.1186/s13006-024-00668-3 PMC11365258

[35] V. Clifford , L. D. Klein , C. Sulfaro , et al., “What Are Optimal Bacteriological Screening Test Cut‐Offs for Pasteurized Donor Human Milk Intended for Feeding Preterm Infants?,” Journal of Human Lactation 37 (2021): 43–51. 33351688 10.1177/0890334420981013

[36] Committee on Nutrition; Section on Breastfeeding; Committee on Fetus and Newborn , “Donor Human Milk for the High‐Risk Infant: Preparation, Safety, and Usage Options in the United States,” Pediatrics 139 (2017): e20163440. 27994111 10.1542/peds.2016-3440

[37] HMBANA , HMBANA Standards for Donor Human Milk Banking: An Overview (Human Milk Banking Association of North America (HMBANA), 2024), https://www.hmbana.org/file_download/inline/95a0362a‐c9f4‐4f15‐b9ab‐cf8cf7b7b866.

[38] S. L. Unger and D. L. O'Connor , “Review of Current Best Practices for Human Milk Banking,” Maternal & Child Nutrition 20, no. Suppl 4 (2024): e13657. 38752309 10.1111/mcn.13657 PMC11184574

[39] N. T. Cacho , N. A. Harrison , L. A. Parker , et al., “Personalization of the Microbiota of Donor Human Milk With Mother's Own Milk,” Frontiers in Microbiology 8 (2017): 1470. 28824595 10.3389/fmicb.2017.01470 PMC5541031

[40] L. F. Stinson , J. Ma , C. T. Lai , A. Rea , S. L. Perrella , and D. T. Geddes , “Milk Microbiome Transplantation: Recolonizing Donor Milk With Mother's Own Milk Microbiota,” Applied Microbiology and Biotechnology 108 (2024): 74. 38194146 10.1007/s00253-023-12965-8 PMC10776751

[41] M. F. Torrez Lamberti , N. A. Harrison , M. M. Bendixen , et al., “Frozen Mother's Own Milk Can be Used Effectively to Personalize Donor Human Milk,” Frontiers in Microbiology 12 (2021): 656889. 33936012 10.3389/fmicb.2021.656889 PMC8079756

[42] Z. Tan , W. Zhong , H. Danielsson , et al., “Immune Development Differs Between Preterm Newborns Fed Mothers' Own Milk and Donor Milk,” iScience 28 (2025): 112918. 40672699 10.1016/j.isci.2025.112918 PMC12266545

[43] K. D. Hendricks‐Muñoz , N. Darwish , N. Chahin , et al., “Successes of a Focused Mothers' Own Milk (MOM) Program in Counteracting Unintended Effects of a Donor Milk Program on MOM Rates at Discharge,” Breastfeeding Medicine 18 (2023): 928–933. 38016149 10.1089/bfm.2023.0147

[44] E. Kontopodi , S. Arslanoglu , U. Bernatowicz‐Lojko , et al., “‘Donor Milk Banking: Improving the Future’. A Survey on the Operation of the European Donor Human Milk Banks,” PLoS One 16 (2021): e0256435. 34411191 10.1371/journal.pone.0256435 PMC8376009

