# Supplementary material for: Human Milk Practices in Swedish Neonatal Units: Results From a Nationwide Survey
Source: Acta Paediatr. 2026 Jan 20;115(5):1048–62. doi: 10.1111/apa.70448 (PMC13063361; doi:10.1111/apa.70448)
Supplement: Supplementary file 1 — Appendix S1: apa70448‐sup‐0001‐AppendixS1.docx. [file APA-115-1048-s001.docx]

| **Map over Swedish healthcare regions (adapted with permission from SNQ)**  **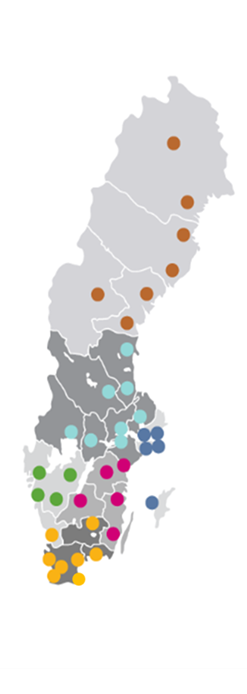**  Brown = North  Light blue = Middle  Dark blue = East  Green = West  Purple = South-east  Yellow = South |
| --- |
