# Supplementary material for: Human Milk Practices in Swedish Neonatal Units: Results From a Nationwide Survey
Source: Acta Paediatr. 2026 Jan 20;115(5):1048–62. doi: 10.1111/apa.70448 (PMC13063361; doi:10.1111/apa.70448)
Supplement: Supplementary file 2 — Appendix S2: apa70448‐sup‐0002‐AppendixS2.docx. [file APA-115-1048-s002.docx]

**Questionnaire**

1. Please indicate what type of care you provide at your hospital: Multiple answer options possible.

- Neonatal intensive care
- Neonatal care
- Couplet-care (mother receives her aftercare within your neonatal care operation)
- Neonatal home care
- Neonatal reception
- Other Please specify

1. How many beds do you have at your hospital? - Number of beds:

- Neonatal intensive care
- Neonatal care

1. Are the parents able to be with their child 24/7? - yes, no
2. From what gestational ages do you accept children for care in the neonatal unit at your hospital according to local guidelines?
3. Do you offer parents support and information on how they can initiate and maintain milk production (lactation support)? -yes, no, sometimes
4. Which staff provide this support? Multiple answer options possible.

- Nurse
- Assistant nurse
- Midwife
- Dietitian
- Doctor
- Other/others Please specify

1. Where do the staff who provide this support mainly work? Multiple answer options possible.

- Neonatal care
- Maternity care
- Other perinatal care

1. Do you inform parents about the importance of breast milk for the baby? – yes, no, sometimes
2. Do you inform parents about the differences between own and donated breast milk? - yes, no, sometimes
3. Do you use a so-called colostrum kit (a starter kit containing information about colostrum, early expression and hand expression as well as, for example, a small collection container and feeding syringe)? -yes, no, sometimes

- If yes, when do parents receive the colostrum kit?
  - Before
  - after
  - could be before of after

1. Do you follow up on how the mother is doing with the expression at any time during the care period? – yes, no, sometimes
2. If yes, how often is the follow-up?
3. If yes, what is included in the follow-up? Multiple answer options possible.

- The mother's emotional experience of expressing
- Amount of breast milk expressed per day
- Another follow-up, please specify

1. If yes, which staff is responsible for the follow-up? Multiple answer options possible.

- Nurse
- Assistant nurse
- Midwife
- Dietitian
- Doctor
- Other, please specify

1. Do you have written guidelines for follow-up of lactation support? – yes, no
2. Is the mother able to express/pump separately and undisturbed in the hospital? – yes, no
3. Are mothers who are expected to express milk for their children offered meals/food on the ward? -yes, no

18. If yes, which meal(s) are offered? Multiple answer options possible.

- Breakfast
- Morning snack
- -Lunch
- Afternoon snack
- Evening meal
- Evening snack

19.Can mothers of children cared for in your facility borrow a breast pump? Multiple answer options possible.

- Yes, manual
- Yes, electric
- No

20. Is the loan of a breast pump free of charge or not for the mother during her child's hospital stay?

- If for a fee, please state the fee/rental cost and whether this is a one-time cost or a weekly/monthly cost

21. Is the rental/loan of a breast pump free of charge or not for the mother after discharge?

- If for a fee, please state the fee/rental cost and whether this is a one-time cost or a weekly/monthly cost

22. What is the maximum period for which a mother can borrow a breast pump from your facility?

23. Is there a breastfeeding clinic at your hospital? -yes, no

24. Do you have continuous training for staff regarding lactation/milk formation and breastfeeding? Multiple answer options possible.

- Yes, for all new employees
- Yes, for all staff annually
- Yes, for all staff every other year
- Yes, for all staff less frequently
- No
- Other, please comment

25. Are all professional categories working within your business offered to participate (including, for example, doctors and physiotherapists and not just nursing staff)?

26. Who conducts the staff training? Multiple answer options possible.

- External lecturer Please specify whether external lecturers can be connected to the academy (e.g. representatives from higher education institutions)
- External lecturer Please specify whether external lecturers may be linked to industry (e.g. representatives from companies that produce nutritional products, breast pumps
- Internal lecturer Please specify whether internal lecturers are linked to neonatal care, OB/GYN care or other perinatal care

27. About mother's own breast milk is frozen, is this given in chronological order (i.e. older expressed milk is given before newer expressed milk)?

28. If the mother has her own milk in the freezer and also has fresh expressed milk, which of these is given first?

- Frozen milk in chronological order is given first
- Frozen milk (independent of chronological order) is given first
- Fresh milk is given first
- A combination of both frozen and fresh mil. Please specify this combination (e.g. if the fresh milk covers the child's prescribed amount of food, then only the fresh milk is given or is this combined with previously expressed frozen milk) and whether the choice of combination is dependent on e.g. gestational age and/or weight"

29. If the mother is on any of the following medications, is her milk given to the baby directly? This question is best answered in consultation with the doctor in the facility. Multiple answer options possible. Please comment on which medication(s), of the following medications, is her milk then given to the baby directly? This question is best answered in consultation with the doctor in the facility. Multiple answer options possible. Please comment on which medication(s), if any dosage, and whether the medicine is allowed or not when relevant. – yes, no, no-frozen and given later, no- the milk is discarded or frozen and given later during care

- Hormone preparations (e.g., thyroid hormone and insulin)
- Local treatment with inhalation preparations for asthma
- Local treatment of skin, nose, and eyes (e.g., corticosteroids and sodium cromoglicate [Lomudal])
- Contraceptives in the form of progestogens
- Pain-relieving medications: temporary treatment, e.g., after a caesarean section (<7 consecutive days)
- Pain-relieving medications: long-term treatment (>7 consecutive days)
- Antihypertensives
- Psychopharmaceuticals
- Herbal medicines

30. Do you have guidelines for breastfeeding mothers who are breastfeeding and drinking alcohol? – yes, no

- If yes, please indicate how many hours after drinking alcohol expressed/pumped breast milk is considered safe to give to the baby

31. Do you provide any information on coffee and caffeine intake for breastfeeding mothers? – yes, no

- If yes, please specify if you have any maximum recommended daily intake of coffee and/or caffeine

32. Please state your guidelines for handling mother's breast milk regarding time aspects for storing the milk and how long it is considered safe to give to the baby before it must be discarded:

- How long can freshly expressed milk be kept in room temperature?
- How long can mother's milk be kept in refrigerator?
- How long can mothers milk be kept in freezer?

33. Which children are offered pasteurized donated breast milk? Multiple answer options possible.

- All children cared for in the neonatal unit (both full-term and premature)
- Only premature babies and babies with low birth weight/growth retarded babiesPlease specify up to which gestational week and/or weight donated breast
- Babies cared for in another ward outside the neonatal unit Please specify which ward(s)
- Other option not covered by above, please specify which babies receive donated breast milk

34. Do you have a priority order for which children receive the donated breast milk with the highest protein content? – yes, no

- If yes, please comment on the priority order and whether this is based on, for example, gestational age and/or weight

35. Please state your guidelines for handling pasteurized donated breast milk regarding time aspects for storing the milk and how long it is considered safe to give to the child before it must be discarded;

36. How long can thawed pasteurised donor milk be kept in room temperature?

37. How long can thawed pasteurised donor milk be kept in refrigerator?

38. How long can thawed pasteurised donor milk be kept in freezer?

39. Please provide your guidelines for handling pasteurized donated breast milk regarding the time aspects of storing the milk and how long it is considered safe to give to the baby before it must be discarded.

40. Do you always discard frozen pasteurized donated breast milk that has passed its best before date/use by date or do you allow it to be given to babies? If you give pasteurized donated breast milk that has passed its expiration date, do you have any time limit after passing date the milk can be given?

41. Do you analyse breast milk for nutritional content (energy, protein, fat and carbohydrates) in your hospital? -yes, no, sometimes

- If sometimes, please specify

42. Do you analyse breast milk for all children cared for in the ward or is it based on gestational age and/or weight? Please specify gestational age and weight limits if these exist. - yes, no

43. How often do you analyse breast milk for nutritional content?

- If your routines regarding this change over time (e.g. analysis is performed weekly for the first four weeks of life and every two weeks thereafter), please specify your routine

44. Indicate the minimum amount of expressed breast milk required for you to prescribe a breast milk analysis:

45. What is your routine regarding collecting a sample of breast milk for analysis of its nutritional content? Multiple answer options possible.

- We collect milk for 24 hours in a row, mix it and take a sample from the mixture
- We take a random sample of mother's milk when an analysis is prescribed
- Both, please comment
- Other, please specify

46. Please provide the name of the analytical equipment you use to analyze the mother's milk and the minimum amount of milk required to run an analysis of the milk's nutritional content;

- Name of equipment
- Minimum amount of milk

47. Do you analyse donated breast milk for nutritional content (energy, protein, fat and carbohydrates) at your hospital? -yes, no, sometimes

- No, the donated breast milk is analysed by the milk bank from which we receive the milk. Please specify from which milk bank
- Sometimes, please specify

48. When is the nutritional analysis of each batch of donated breast milk performed? A batch is defined as the amount of donated breast milk from one and the same donor that is pasteurized at the same time. All donated breast milk from a batch therefore has the same identification code.

49. What protein value from the nutritional analysis of the mother's own breast milk do you use for nutritional value calculations?

50. What protein value from the nutritional analysis of donated breast milk do you use for nutritional value calculations?

51. Which children receive fortified breast milk in your practice? Do you have any limits based on gestational age and/or weight?

52. What is your routine for fortification of breast milk?

53. If a nutritional analysis of the breast milk is not available, do you still fortify the milk?

54. What is the fortification procedure if a child receives both own and donated breast milk during the same 24 hours?

55. Do you prescribe fortification of breast milk based on results from current breast milk analysis? Current breast milk analysis refers to the nutritional value analysis that corresponds to the pumping date(s) of the milk given to the child.

56. Do you use any program to calculate the nutritional content of the breast milk you give to the children (including, for example, fortifications and other nutrition)?

- If yes, please specify the name of the program

57. Which professional groups work in the milk kitchen preparing meals, enriching breast milk and mixing formula for the children?

58. Does this staff also work in the care of the patients?

59. Does the staff in the milk kitchen work mixed with caring for children during the same day?

60. Please indicate which part(s) of the handling and pasteurization process of donated breast milk you perform yourself. The handling process involves, for example, contacting donors, health declaration and blood sampling for donors, lending breast milk pumps to donors, and receiving donated breast milk from donors. The pasteurization process involves pasteurization and bacteriological sampling, as well as performing nutritional analysis, of the donated breast milk.

61. How are donors recruited? Multiple answer options possible.

- Visit to a breast milk center/milk bank
- Neonatal unit
- Postnatal ward
- Return visit clinic
- Child health centre
- Maternal health care
- Telephone
- Social media
- Advertising, please specify where the advertising occurs (e.g. maternity hospital, neonatal care, child health centre, maternal health care, non-profit associations, newspapers, etc.)
- Other Please specify

62. Do you have a time limit for how long a woman can donate breast milk?

- If yes, please specify how long and whether the time from parturition or from when the blood sample was taken prior to donation

63. Have you had to refuse a breast milk donor and if so, approximately how many times per year?

64. How does the milk get to you? Multiple possible answers.

- The donors come and leave the milk themselves
- Via home care
- Via transport, please specify
- Other, please specify

65. Do you provide the donors with a breast pump? Multiple possible answers.

- Yes, manual
- Yes, electric
- No

66. Is the loan of a breast pump free of charge or not for the donor?

- If for a fee, please specify the fee/rental cost and whether this is a one-off cost or a weekly/monthly cost

67. Is collected drip milk accepted for donation (milk that leaks between pumping or from the other breast during expression and is collected in a milk collector)? – yes, no

68. Do donors receive compensation? -yes, no

- If yes, please specify the type of compensation, how much and how often the compensation is given

68. How many employees work with handling donated breast milk?

69. Which professional groups work with handling donated breast milk?

70. Do these personnel also work in the care of patients? -yes, no

71. Does the staff work with the handling of donated breast milk mixed with the care of children during the same day? – yes, no

72. How many hours per week do you work with the handling of donated breast milk?

73. What is included in the health declaration? Please attach a copy of the health declaration here. Otherwise, please state what the health declaration contains in the comments box below.

74. What is included in the health declaration? Please attach a copy of the health declaration here. Otherwise, please state what the health declaration contains in the comments box below.

- Comment

75. Is a donor approved if he/she is taking any of the following medications? This question is best answered in consultation with the doctor in the facility. Multiple answer options are possible. Please comment on which medication(s), any acceptable dosage limits, and whether the medication is allowed or not when relevant.- yes, no

- Hormone preparations (e.g., thyroid hormone and insulin)
- Local treatment with inhalation preparations for asthma
- Local treatment of skin, nose, and eyes (e.g., corticosteroids and sodium cromoglicate [Lomudal])
- Contraceptives in the form of progestogens
- Pain-relieving medications
- Antihypertensives
- Psychopharmaceuticals
- Herbal medicines

76. Is a donor approved if they consume alcohol during the donation period?

- If yes, please specify how many hours after consuming alcohol the woman is allowed to donate breast milk

77. Do you have any guidelines regarding coffee and caffeine intake for donors?

- If yes, please specify if you have any stated maximum recommended daily intake of coffee and/or caffeine

78. Which of the following blood tests are taken when recruiting donors.

Tick which of the following blood tests are taken during donor recruitment.

- HIV-1
- HIV-2
- HTLV-I
- HTLV-II
- Hepatitis B
- Hepatitis C
- Syphilis
- Other Please specify

79. If a woman has donated breast milk for three months and wishes to continue donating her breast milk, is a new blood test prescribed?

- If yes, please specify which blood samples are taken and how often you take blood samples from women who donate

80. Are vegan women allowed to donate breast milk?

81. Do you require vegan donors to take extra supplements?

- If yes, please specify which supplements

82. Where is the breast milk centre/milk bank that works to pasteurize donated breast milk located?

83. How many employees work to pasteurize donated breast milk?

84. Which professional groups work to pasteurize donated breast milk?

85. Does this staff also work in patient care?

86. Does the staff work to pasteurize donated breast milk mixed with caring for children on the same day?

87. How many hours per week do you work pasteurizing donated breast milk?

88. Do you supply other departments and/or hospitals with donated breast milk? Multiple answer options possible.

- Yes, other departments in our hospital, please specify which departments
- Yes, other hospitals within the healthcare region
- Yes, other hospitals outside the healthcare region
- No

89. Is all donated breast milk pasteurized?

- No, please state when donated breast milk is not pasteurized

90. Which pasteurization method is used (e.g. Holder pasteurization which involves heating the milk to 62.5 °C for 30 minutes)?

91. How many batches are pasteurized at each pasteurization event? A batch is defined as the amount of donated breast milk from a single donor that is pasteurized at the same time. All donated breast milk from a batch therefore has the same identification code.

92. Do you mix breast milk from different donors (so-called breast milk pooling)?

- If yes, please state the reason for pooling breast milk
- If sometimes, please state the reason for pooling breast milk

93. What is your routine for bacteriological sampling of the same batch of donated breast milk? A batch is defined as the amount of donated breast milk from the same donor that is pasteurized at the same time. All donated breast milk from a batch therefore has the same identification code.

94. How are the bacterial tests performed?

- Scratch test on frozen milk
- On fresh or thawed milk
- Other, please specify

95. How are the bacterial tests performed?

- Random test from one of the cans/bottles in a batch
- A mixture of all milk from a batch
- Both, please specify
- Other, please specify

96. When are the bacterial samples taken?

- Before pasteurisation
- After pasteurisation
- Before and after pasteurisation

97. Is the donated breast milk evaluated for bacterial content according to the following guidelines?

- No potentially pathogenic bacteria such as beta-hemolytic streptococci group A, C or G, streptococci group B, Listeria or Salmonella
- < 107 cfu/L of Staphylococcus aureus
- < 107 cfu/L of gram-negative rods belonging to Enterobacteriaceae
- < 107 cfu/L of Pseudomonas aeruginosa or other Pseudomonas species
- < 107 cfu/L of Stenotrophomonas maltophilia
- < 107 cfu/L of Acinetobacter
- Aerobic bacteria, e.g. coagulase-negative staphylococci (CNS) or alpha-streptococci

98. Is the donated breast milk evaluated for any other bacteria?

- If yes, please state which bacteria and at what threshold values ​​the milk is positive

99. What analysis method is used to determine bacterial levels?

100. In case of excess donated breast milk, do you share it with other hospitals and/or healthcare regions? – yes, no, sometimes

101. When you sell breast milk to other hospitals and/or healthcare regions, what do you sell it for? Please state the price/litre.

102. Have you had to refuse breast milk donations due to restrictions within the organization? For example, not enough refrigerator and freezer space or not enough time to work with handling and pasteurizing donated breast milk. – yes, no

- If yes, please specify

103. Do you want to add or clarify anything?
